# A systematic review of diagnostic methods to differentiate acute lung injury/acute respiratory distress syndrome from cardiogenic pulmonary edema

**DOI:** 10.1186/s13054-017-1809-8

**Published:** 2017-08-25

**Authors:** Kosaku Komiya, Tomohiro Akaba, Yuji Kozaki, Jun-ichi Kadota, Bruce K. Rubin

**Affiliations:** 1 Children’s Hospital of Richmond at Virginia Commonwealth, Richmond, VA 23298 USA; 2 Respiratory Medicine and Infectious Diseases, Oita University Faculty of Medicine, 1-1 Idaigaoka, Hasama-machi, Yufu, Oita 879-5593 Japan; 3 Clinical Research Center of Respiratory Medicine, Tenshindo Hetsugi Hospital, 5956 Nihongi, Nakahetsugi, Oita 879-7761 Japan

**Keywords:** Heart failure, Acute respiratory failure, Pulmonary edema, Biomarkers

## Abstract

**Background:**

Discriminating acute lung injury (ALI) or acute respiratory distress syndrome (ARDS) from cardiogenic pulmonary edema (CPE) is often challenging. This systematic review examines studies using biomarkers or images to distinguish ALI/ARDS from CPE.

**Methods:**

Three investigators independently identified studies designed to distinguish ALI/ARDS from CPE in adults. Studies were identified from PubMed, and the Cochrane Central Register of Controlled Trials database until July 3, 2017.

**Results:**

Of 475 titles and abstracts screened, 38 full texts were selected for review, and we finally included 24 studies in this systematic review: 21 prospective observational studies, two retrospective observational studies, and one retrospective combined with prospective study. These studies compared various biomarkers to differentiate subjects with ALI/ARDS and in those with CPE, and 13 calculated the area under the receiver operator characteristic curve (AUC). The most commonly studied biomarker (four studies) was brain natriuretic peptide (BNP) and the discriminatory ability ranged from AUC 0.67–0.87 but the timing of measurement varied. Other potential biomarkers or tools have been reported, but only as single studies.

**Conclusions:**

There were no identified biomarkers or tools with high-quality evidence for differentiating ALI/ARDS from CPE. Combining clinical criteria with validated biomarkers may improve the predictive accuracy.

## Background

Differentiating between cardiogenic pulmonary edema (CPE) and acute lung injury (ALI) or acute respiratory distress syndrome (ARDS) is challenging in the early stages of illness [1]. The most widely accepted definition of ALI/ARDS had been based on the American-European Consensus Conference (AECC) definition, of acute onset respiratory failure with bilateral infiltrates on chest radiograph, and pulmonary capillary wedge pressure (PCWP) <18 mmHg, or absence of elevated left atrial pressure [2]. However, pulmonary artery catheterization is rarely used in clinical practice because clinical estimation of PCWP is invasive, costly, and does not aid in the diagnosis of ALI/ARDS [3–7]. There were potential inconsistencies in this definition, including a lack of explicit criteria for defining acute respiratory failure, the sensitivity of the PaO2/FiO2 (P/F) ratio to ventilator settings, poor reliability of the chest radiograph criteria, and difficulties distinguishing ARDS from CPE including that these diagnoses can coexist [8, 9]. Based on these limitations, the Berlin definition for ARDS was published in 2012 and is reported to have better predictive validity for mortality, than this earlier definition [8]. Pulmonary capillary wedge pressure measurement was removed from this definition. Patients were presumed to have ARDS if they had respiratory failure not fully explained by cardiac failure or fluid overload as judged by the treating physician using all available data.

In current practice and most clinical studies, ALI/ARDS is usually differentiated from CPE by the clinical circumstances and by physical findings, but this distinction is often made only by post hoc review after patient’ discharge or death, and is often based on the response to therapy [10, 11]. The ARDS Clinical Trial Network reported that fluid management to decrease cardiogenic fluid retention and the effects of lung permeability and edema, will shorten the duration of mechanical ventilation and intensive care without increasing nonpulmonary organ failure [12]. The differentiation between ALI/ARDS and CPE is important in order to avoid delaying treatment of fluid retention and avoiding unnecessary testing [13]. Several biomarkers to distinguish ALI/ARDS from CPE have been reported. The aim of this systematic review was to review published studies of potential biomarkers to distinguish ALI/ARDS from CPE.

## Methods

This systematic review was conducted using the Preferred Reporting Items for Systematic Reviews and Meta-Analyses (PRISMA) and the Meta-analysis of Observational Studies in Epidemiology (MOOSE) guidelines [14].

### Search criteria

We included prospective or retrospective cohort studies written in English, which evaluated biomarkers or images for differentiating ALI/ARDS from CPE in adults. Studies that did not refer ALI/ARDS based on the AECC or the Berlin definition were excluded from this systematic review [2, 8]. We identified studies from the PubMed database using the search terms: “acute lung injury [All Fields] OR acute respiratory distress syndrome [All Fields] OR pneumonia [All Fields] AND cardiogenic pulmonary edema [All Fields] OR hydrostatic pulmonary edema [All Fields] OR ARDS diagnostics [All Fields] OR decompensated heart failure [All Fields]”, and from the Cochrane Central Register of Controlled Trials database using the search terms: “acute lung injury AND cardiogenic pulmonary edema”, “acute respiratory distress syndrome AND cardiogenic pulmonary edema”, and “pneumonia AND cardiogenic pulmonary edema” (accessed on July 3, 2017). All included studies focused on distinguishing “pure” ALI/ARDS from “pure” CPE. Mixed cases were excluded from analysis. Studies published only in abstract form were excluded. Full texts of articles were further evaluated by three investigators (KK, TA, and YK).

### Data extraction

We extracted the following information from included studies: study design, sample size, diagnostic methods of ALI/ARDS or CPE, assessed markers, mean value of the markers in ALI/ARDS or CPE, the area under the receiver operator characteristic curve (AUC), and specificity and sensitivity for ALI/ARDS or CPE at a cutoff.

### Assessing risk of bias

The risk of bias in the included studies was assessed according to the recommendations outlined in the Cochrane Handbook for Systematic Reviews of Interventions Version 5.1.0. and MOOSE guidelines for the following items: selection, performance, detection, attrition, and publication bias [14]. Each study included in this review was assessed for quality as good, moderate, or poor based on biases using the modified Hayden’s criteria [15], which included source population, sample size, inclusion criteria, how to determine the final diagnosis of ALI/ARDS or CPE, and analysis providing sufficient presentation of data. Three investigators independently determined the quality based on these points. Disagreements among the investigators were resolved by review of the assessments to reach consensus.

## Results

### Database search and characteristics of included studies

**Fig. 1:**
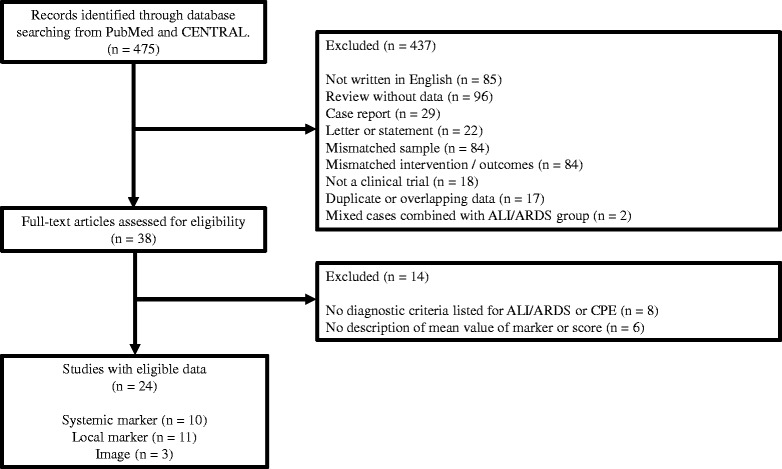
Flow diagram of the study selection

We identified 475 studies through PubMed and CENTRAL databases, and then excluded 437 studies as the abstract did not meet the inclusion criteria. We excluded 14 of the remaining 38 records after retrieving and inspecting the full text (Fig. 1).

We finally included 24 studies in this systematic review: ten studies using systemic biomarkers which were measured in plasma [the quality of these studies by the modified Hayden’s criteria was, good (n = 6) [10, 16–20], moderate (n = 1) [21], and poor (n = 3) [22–24]], and 11 studies using “lung-specific” biomarkers measured in bronchoalveolar lavage (BAL) or pulmonary edema fluid [quality was good (n = 2) [25, 26] and poor (n = 9) [27–35]], three studies of chest ultrasonography or computed tomography (CT) [good (n = 1) [36] and moderate (n = 2) [37, 38]]. Most were reported as prospective cohort studies but two studies were retrospective and one study combined a retrospective cohort with a prospective cohort. These studies were published from the USA (n = 10), Japan (n = 5), Germany (n = 2), Australia (n = 2), China (n = 2), Italy (n = 1), Belgium (n = 1), The Netherlands (n = 1), Taiwan (n = 1), and Switzerland (n = 1).

Sixteen studies diagnosed either ALI/ARDS or CPE using clinical information [10, 16–18, 21, 22, 24–27, 29, 30, 34, 36–38], and in eight studies the final diagnosis was confirmed by at least two independent reviewers [10, 16–18, 21, 26, 36, 37]. Six studies [19, 20, 23, 32, 33, 35] used the results of PCWP by pulmonary artery catheterization and two studies used the edema fluid protein/plasma protein ratio to differentiate ALI/ARDS from CPE [28, 31].

### Systemic biomarkers

Nine of ten studies that evaluated systemic markers to distinguish ALI/ARDS from CPE assessed predictive power using AUC. Brain natriuretic peptide (BNP) was the most commonly assessed biomarker (Table 1). The discriminatory ability to differentiate CPE from ARDS varied among four studies with AUC, 0.67–0.83. The levels of plasma CRP in patients with ALI/ARDS were significantly higher than those with CPE [18, 33]. In these studies, subjects thought to have both ALI/ARDS and CPE were explicitly excluded from the analysis. Komiya and colleagues showed that when C-reactive protein was used to differentiate CPE from ALI/ARDS, the AUC was as good as BNP, and the AUC when the combination of BNP and CRP was used to differentiate CPE from ALI/ARDS was significantly higher than either BNP or CRP alone.

**Table 1:** Systemic markers differentiating ALI/ARDS from CPE

Study	Design	No. of ALI/ARDS vs CPE	marker	Timing of measurement	Mean value ± SD/SEM or median (IQR or range) in ALI/ARDS	Mean value ± SD/SEM or median (IQR or range) in CPE	Unit	*p* value	AUC	SD or 95% CI	Cutoff	For	Specificity (%)	Sensitivity (%)
Markers of cardiomyocyte stress														
Bajwa, 2013***	Retrospective	826 vs 87	ST-2	The day of enrollment	534 (IQR325.0–724.9)	43 (IQR26.4–78.6)	ng/mL	<0.0001	0.98	n.d. (*p* <0.0001)	142	ALI/ARDS	91	94
Komiya, 2011***	Prospective	53 vs 71	BNP	Within 2 h after arriving	202 (IQR95–439)	691 (IQR416–1194)	pg/mL	<0.001	0.831	0.759–0.904	500	CPE	83.1	69
			CRP	at ER	119 (IQR62–165)	8 (IQR2–42)	mg/L	<0.001	0.887	0.826–0.948	50	ALI/ARDS	69.8	59.2
			BNP + CRP		n.d.	n.d.	n.d.	n.d.	0.931	0.884–0.978	n.d.	n.d.	n.d.	n.d.
Levitt, 2008**	Prospective	33 vs 21	BNP	Within 48 h of ICU adm.	369 (IQR87–709)	600 (IQR352–1300)	pg/mL	0.04	0.67	0.52–0.81	100	ALI/ARDS	95.2	27.3
Karmpaliotis, 2007***	Prospective	51 vs 23	BNP	Not stated	325 (IQR82–767)	1260 (IQR541–2020)	pg/mL	0.0001	0.79	n.d.	200	ALI/ARDS	91	40
Rana, 2006***	Retrospective + Prospective	131 vs 73	BNP	Median 3 h after diagnosis	344 (IQR122–745)	759 (IQR378–1320)	pg/mL	<0.001	0.71	n.d.	250	ALI/ARDS	90	40
Other circulating markers														
Lin Q, 2013***	Prospective	78 vs 28	Plasma HBP	At enrollment	17.15 (IQR11.95–24.07)	9.50 (IQR7.98–12.18)	ng/mL	<0.001	0.851	±0.040	11.55	ALI/ARDS	78.2	75
Lin Q, 2012***	Prospective	87 vs 34	Copeptin	At enrollment	52.53 (IQR29.81–91.43)	25.14 (IQR21.04–34.26)	pmol/L	<0.001	0.823	±0.038	40.11	ALI/ARDS	88.2	60.9
Arif, 2002*	Prospective	11 vs 12	Transferrin in plasma	Within 72 h of ICU adm.	1.0 (range 0.5–1.5)	2.1 (range 1.5–2.7)	g/L	<0.001	0.98	n.d.	1.5	ARDS	87	100
			TP in plasma		49 (range 41–59)	63 (range 51–69)	g/L	<0.001	0.95	n.d.	59	ARDS	75	100
			Alb in plasma		25 (range17–34)	30 (range 25–43)	g/L	NS	0.8	n.d.	24	ARDS	100	45
			Pulmonary leak index		32.3 (range23.0–54.4)	10.1 (range 4.4–16.2)	X10^-3/m	<0.001	1	n.d.	16.3	ARDS	100	100
Shih, 1997*	Prospective	13 vs 5	MAA in serum	Not stated	53.8 ± 6.6 SEM	9.0 ± 3.1 SEM	ng/mL	<0.05	n.d.	n.d.	n.d.	n.d.	n.d.	n.d.
Backer, 1997*	Prospective	43 vs 9	AVLAC	Not stated	0.20 ± 0.230 SD	0.139 ± 0.176 SD	mEq/L	<0.001	n.d.	n.d.	n.d.	n.d.	n.d.	n.d.

*Alb* albumin, *ALI* acute lung injury, *ARDS* acute respiratory distress syndrome, *AUC* area under the curve, *AVLAC* arteriovenous differences in lactate, *BNP* brain natriuretic peptide, *CPE* cardiogenic pulmonary edema, *CRP* C-reactive protein, *ER* emergency department, *HBP* heparin-binding protein, *MAA* mucin-associated antigen, *n.d.* not described, *SP-A* surfactant Protein-A, *SP-B* surfactant protein-B, *SEM* standard error of the mean, *SD* standard deviation, *ST-2* suppression of tumorigencity-2, *TP* total protein

***good, **moderate, or *poor quality assessed based on the modified Hayden’s criteria

The plasma soluble suppression of tumorigenicity-2 [20], heparin-binding protein [39], and copeptin [16] were evaluated in single studies that showed high predictive value for differentiating ALI/ARDS from CPE. Arif and colleagues reported that pulmonary leak index was significantly higher in ARDS than in CPE patients and the AUC for ARDS was 0.98 for transferrin, 0.95 for total protein, and 0.80 for albumin levels in plasma [22]. Other studies compared mean value of mucin-associated antigen in serum, or arteriovenous differences in lactate between ALI/ARDS and CPE but the sample size for each of these studies was small, and the methods used as the standard for diagnosis were unclear.

### Lung biomarkers

Only one of the 11 studies that evaluated “lung-specific” biomarkers used AUC to evaluate their ability to distinguish ALI/ARDS from CPE (Table 2). Ware and colleagues showed that the fluid-to-plasma protein ratio had a high AUC and good sensitivity and specificity for differentiating ALI from CPE, and that a fluid to plasma ratio >0.65 was associated with higher mortality and more days requiring mechanical ventilation [25]. Schutte and colleagues reported that the protein concentration in BALF from ALI/ARDS subjects was higher than in CPE [33]. In two studies, surfactant apoprotein (SP)-A was significantly greater in BALF from subjects with CPE compared to those with ALI/ARDS [32, 35]. Laminin gamma-2 fragments are parts of laminin-5, which is a cellular adhesion molecule expressed solely by epithelium, and promotes epithelial cell migration and repair of injured epithelium [40]. The concentration of these fragments in epithelial lining fluid from subjects with ALI/ARDS was significantly higher than those with CPE, and the concentration of laminin gamma-2 fragments at 5 days after onset also was associated with mortality [27].

**Table 2:** Localized markers for differentiating ALI/ARDS from CPE

Study	Design	No. of ALI/ARDS vs CPE	Marker	Timing of measurement	Mean value ± SD/SEM or median (IQR or range) in ALI/ARDS	Mean value ± SD/SEM or median (IQR or range) in CPE	Unit	*p* value	AUC	95% CI	Cutoff	For	Specificity (%)	Sensitivity (%)
Markers of airway epithelium injury														
Katayama, 2010*	Prospective	21 vs 11	Laminin gannma2 in ELF	Not stated	6034 ± 6245 SD	1237 ± 807 SD	ng/mL	<0.02	n.d.	n.d.	n.d.	n.d.	n.d.	n.d.
			Laminin gannma2 in plasma		147 ± 83 SD	35 ± 14 SD	ng/mL	<0.0001	n.d.	n.d.	n.d.	n.d.	n.d.	n.d.
Kropski, 2009*	Prospective	23 vs 9	CCP 16 in PEF	Within 24 h of diagnosis	1950 (IQR1780–4024)	4835 (IQR2006–6350)	ng/mL	0.044	n.d.	n.d.	n.d.	n.d.	n.d.	n.d.
			CCP 16 in serum		22 (IQR9–44)	55 (IQR18–123)	ng/mL	0.053	n.d.	n.d.	n.d.	n.d.	n.d.	n.d.
Chandel, 2009*	Prospective	15 vs 5	KGF in BAL	Within 48 h after intubation	1.2-fold ±0.12 SD	2.4-fold ±0.48 SD	fold	<0.01	n.d.	n.d.	n.d.	n.d.	n.d.	n.d.
Newman, 2000*	Prospective	15 vs 12	HTI56 in PEF	Within 15 min after intubation	1451 ± 6 727	335 ± 123	ug/mL	<0.0001	n.d.	n.d.	n.d.	n.d.	n.d.	n.d.
			HTI56 in plasma		217 ± 79	335 ± 123	ug/mL	<0.05	n.d.	n.d.	n.d.	n.d.	n.d.	n.d.
Shimura,1996*	Prospective	5 vs 11	SP-A in sputum	Not stated	311 ± 47 SEM	1324 ± 197 SEM	ug/mL	<0.001	n.d.	n.d.	n.d.	n.d.	n.d.	n.d.
Gunther, 1996*	Prospective	15 vs 13	SP-A in BAL	Within 72 h after intubation	849 ± 96 SEM	1013 ± 111 SEM	ng/mL	n.d.	n.d.	n.d.	n.d.	n.d.	n.d.	n.d.
			SP-B in BAL		867 ± 131 SEM	628 ± 42 SEM	ng/mL	n.d.	n.d.	n.d.	n.d.	n.d.	n.d.	n.d.
Protein ratio of fluid to plasma														
Ware, 2010***	Prospective	209 vs 147	Fluid-to-plasma protein ratio	Not stated	0.89 ± 0.36 SD	0.53 ± 0.21 SD	n.d.	<0.001	0.84	0.79-0.88	0.65	ALI/ARDS	81	81
Colucci, 2009*	Prospective	18 vs 9	Protein ratio of fluid by s-Cath to plasma	Within 1–4 h after intubation	Prim. 0.32 ± 0.42 SD, Sec. 0.81 ± 0.33 SD	0.20 ± 0.19 SD	n.d.	0.002 in Sec. vs CPE	n.d.	n.d.	n.d.	n.d.	n.d.	n.d.
Schutte, 1996*	Prospective	12 vs 6	Protein in BAL	Within 72 h after intubation	671 ± 256 SEM	291 ± 81 SEM	ug/mL	n.d.	n.d.	n.d.	n.d.	n.d.	n.d.	n.d.
			PMN in BAL		16.1 ± 5.8 SEM	0.4 ± 0.11 × 10^6	/mL	n.d.	n.d.	n.d.	n.d.	n.d.	n.d.	n.d.
			Serum CRP*		235 ± 33 SEM	93 ± 25 SEM	mg/L	n.d.	n.d.	n.d.	n.d.	n.d.	n.d.	n.d.
Miller, 1996*	Prospective	27 vs 8	Protein in PEF/plasma	Within 30 min after intubation	0.90 ± 0.09 SEM in w/o sepsis, 0.84 ± 0.16 SEM in w sepsis,	0.48 ± 0.12 SEM	Ratio	<0.05	n.d.	n.d.	n.d.	n.d.	n.d.	n.d.
Others														
Kushimoto, 2012***	Prospective	207 vs 26	EVWI	Not stated	18.5 ± 6.8 SD	14.4 ± 4.0 SD	mL/kg	<0.01	n.d.	n.d.	n.d.	n.d.	n.d.	n.d.
			PVPI		3.2 ± 1.4 SD	2.0 ± 0.8 SD	n.d.	<0.01	0.886	0.836-0.953	2.6–2.85	ALI/ARDS	0.9	0.95

*ALI* acute lung injury, *ARDS* acute respiratory distress syndrome, *AUC* area under the curve, *BAL* bronchoalveolar lavage, *CPE* cardiogenic pulmonary edema, *CCP* Clara cell protein, *CRP* C-reactive protein, *ELF* epithelial lining fluid, *EVWI* extravascular lung water index, *HTI56* human type I cell-specific apical membrane protein, *KGF* keratinocyte growth factor, *n.d.* not described, *n.s.* not significant, *PEF* pulmonary edema fluid, *PMN* polymorphonuclear neutrophils, *PVPI* pulmonary vascular permeability index, *s-Cath* suction catheter, *SD* standard deviation, *SEM* standard error of the mean, *SP-A* surfactant Protein-A, *SP-B* surfactant protein-B

***Good, or *poor quality assessed based on biases using the modified Hayden’s criteria

### Imaging studies

Copetti and colleagues evaluated the ability of chest ultrasound to detect characteristic signs of ALI/ARDS vs CPE [38] (Table 3). During normal breathing, sonography can detect the lung moving or “sliding” along the pleura, but this sliding is impaired when there are inflammatory adhesions. While subjectively, normal lung sliding is seen in subjects with CPE, it is absent or decreased in subjects with ALI/ARDS. “B lines” on chest sonography (distinct from Kerly B lines on plain radiography, and previously called comet-tail artifacts), are generated from the thickened interlobular septa (e.g., seen in interstitial edema) at the lung wall interface [41]. Sekiguchi and colleagues reported that a higher “B-line ratio” (proportion of chest zones with positive B lines relative to all zones examined) was specific for the diagnosis of CPE, and that findings of a left-sided pleural effusion >20 mm, moderate or severe left ventricle dysfunction, and minimal diameter of inferior vena cava >23 mm were helpful to distinguish CPE from ALI/ARDS using a derived, simplified prediction score as shown in Table 3 [36]. Some features on chest CT were reported to better differentiate ARDS from CPE. Small ill-defined opacities, defined as patchy areas of ground-glass attenuation or airspace consolidation, and left-dominant pleural effusion had high specificity for ALI/ARDS in a single-center retrospective study [37].

**Table 3:** Imaging to differentiate ALI/ARDS from CPE

Study	Design	No. of ALI/ARDS vs CPE	Marker	Timing of measurement	AUC	95% CI	Cutoff	For	Specificity (%)	Sensitivity (%)
Sekiguchi, 2015***	Prospective	42 vs 59	Combined cardiac and thoracic ultrasonography	Within 6 h of diagnosis						
			Number of chest zones with positive B-lines		0.82	0.75–0.88	3	Miscellaneous causes	42 (95% CI 32–52)	100 (95% CI 89–100)
			Score; left pleural effusion >20 mm (+4), Moderate or severe LV dysfunction (+3), and IVC minimal diameter < =23 mm (-2).		0.79	0.70–0.87	> = 6	CPE	98 (95% CI 87–100)	39 (95% CI 26–52)
Komiya, 2013**	Retrospective	20 vs 41	Chest CT	Within 2 hours of arrival at ED						
			Upper-lobe-predominant GGA					CPE	95	48.8
			Central-predominant GGA					CPE	90	58.5
			Central airspace consolidation					CPE	90	56.1
			Small ill-defined opacities					ARDS	87.8	35
			Left-dominant pleural effusion					ARDS	95.1	25
Copetti, 2008**	Prospective	18 vs 40	Chest sonography	Not stated						
			Alveolar-interstitial syndrome					ALI/ARDS	0	100
								CPE	0	100
			Pleural line abnormalities					ALI/ARDS	45	100
								CPE	0	25
			Reduction or absence of lung sliding					ALI/ARDS	100	100
								CPE	0	0
			Spared areas					ALI/ARDS	100	100
								CPE	0	0
			Consolidations					ALI/ARDS	100	83.3
								CPE	0	0
			Pleural effusion					ALI/ARDS	5	66.6
								CPE	33.3	95
			Lung pulse					ALI/ARDS	100	50
								CPE	50	0

*ALI* acute lung injury, *ARDS* acute respiratory distress syndrome, *AUC* area under the curve, *B-lines* vertical narrow based lines arising from the pleural line to the edge of the ultrasound screen, *CT* computed tomography, *CPE* cardiogenic pulmonary edema, *GGA* ground-glass attenuation, *ED* emergency department, *IVC* inferior vena cava, *LV* left ventricular

***Good, or **moderate quality assessed based on biases using the modified Hayden’s criteria

Miscellaneous including unilateral pneumonia, atelectasis, COPD, pulmonary embolism or pneumothorax

## Discussion

We systematically reviewed serum and pulmonary biomarkers, and imaging used to differentiate ALI/ARDS from CPE. BNP, released from ventricular cardiomyocytes in response to both ventricle volume expansion and pressure overload, was the most commonly evaluated biomarker; but the predictive ability was variable. While two studies measured BNP levels early in the clinical presentation, and these showed a good discriminatory ability [18, 19], other studies allowed BNP to be tested up to 3 hours (IQR 0.5–14) [10] or 48 hours after presentation and these were less able to distinguish ARDS/ALI from CPE [21]. BNP is known to decrease after treatment for heart failure [42] and this could explain the higher discriminatory ability before starting treatment. Replicate and prospective studies with consistent timing of measurements are required in order to improve the quality of evidence.

As well, the subjects in the study by Levitt [21] were younger than in the other studies [10, 18, 19] and in the younger subjects BNP was a less sensitive biomarker. Because elderly patients may respond less well to diuretics, ACE inhibitors, and inotropic agents compared to younger patients [43], the younger patients may also respond to treatment more rapidly. Renal failure often accompanies severe sepsis and ARDS and this can increase BNP despite normal cardiac function [44]. Due to these different patient characteristics in each study, we did not collect these raw data to combine for a meta-analysis.

CRP is widely used as a marker of systemic inflammation, and in one study by the authors of this review; AUC when CRP was used to differentiate ALI/ARDS from CPE was as good as when BNP was used [18]. While BNP levels can increase in some conditions such as renal failure or sepsis despite normal cardiac function [44], CRP is not directly influenced by cardiac function. CRP combined with BNP may have greater discriminatory ability than either BNP or CRP alone [18].

Plasma soluble suppression of tumorigenicity-2, an IL-1 receptor family member which is a mediator of inflammation and immunity, showed excellent discrimination [20]. Heparin-binding protein is an antimicrobial protein stored in neutrophil granules, and it induces cytoskeletal rearrangement of endothelial cells, which leads to breakdown of cell barriers and an increase in macromolecular efflux [39]. Copeptin, the C-terminal portion of the arginine vasopressin precursor, is secreted together with arginine vasopressin precursor from the neurohypophysis. This secretion is thought to reflect the inflammatory cytokine response and the presence of hemodynamic and osmoregulatory disturbances [45, 46]. These biomarkers appeared to be robust in discriminating ALI/ARDS from CPE in single studies; so validation in replicate studies will be necessary.

Sample size in each study for lung-specific biomarkers was small compared with studies of serum biomarkers, and airway sampling by BAL may be difficult in the emergency department setting. When pulmonary edema is present, pulmonary edema fluid can be obtained by inserting a suction catheter into an endotracheal tube until frothy fluid is obtained by suctioning [25]. The pulmonary edema fluid-to-plasma protein ratio has been studied for decades as a tool to differentiate pulmonary permeability edema from hydrostatic edema [47].

Combining cardiac and thoracic ultrasonography could help to determine the cause of acute pulmonary edema [36]. However, these techniques are operator-skill dependent. Chest CT may be better at discriminating ALI/ARDS from CPE than chest radiography; although CT is rarely performed for acute respiratory failure in the emergency department setting. Milne and colleagues performed an independent two-observer study of chest radiographs from 61 subjects with cardiac disease, and 28 with capillary permeability edema, not described as ALI or ARDS [48]. The overall accuracy for distinguishing capillary permeability edema from cardiac edema was 91%. Another study reported that 87% of subjects with hydrostatic edema but only 60% of those with increased permeability edema were correctly identified in critical ill patients [49]. It is controversial if chest radiography can be recommended to differentiate the type of pulmonary edema.

A limitation of studies focusing on biomarkers or images for discriminating ALI/ARDS from CPE is that these can coexist [8]. Some degree of hydrostatic edema is present in many cases of ALI/ARDS, in fact the pulmonary capillary wedge pressure is reported to be elevated in 30% of ARDS patients [12]. Schmickl and colleagues developed a decision support algorithm to distinguish CPE from ARDS based on clinical data [11]. However, while all studies included in this systematic review specifically excluded mixed cases of ALI/ARDS with CPE, these authors included these cases in the ALI/ARDS group. Protein biomarkers such as BNP showed no statistically significant difference when comparing “pure” CPE with serum from subjects with ALI/ARDS both with and without CPE. BNP levels were elevated for both ALI/ARDS with and without CPE and pure CPE (708 pg/mL vs 749 pg/mL; *p* = 0.18). Strict fluid management that addresses cardiogenic pulmonary edema and pulmonary permeability edema increases ventilator-free days [12]. This suggests that biomarkers like BNP could be useful for differentiating CPE from ALI/ARDS and for initiating fluid restriction and diuretics early to decrease the risk of CPE.

We identified that some biomarkers, e.g., soluble suppression of tumorigencity-2, BNP plus CRP, heparin-binding protein, and plasma transferrin had high AUCs for differentiating ALI/ARDS from CPE, but these were each only assessed in a single study. All studies compared the biomarker measurement to the clinical diagnosis, but no study compared accuracy of the clinical diagnosis alone to that of clinical diagnosis plus biomarkers. Since ALI/ARDS and CPE can certainly co-exist, biomarkers need to be considered to evaluate the relative role of CPE in contributing to the morbidity of ALI/ARDS.

Because there are no accepted criteria for differentiating CPE from ALI/ARDS at the time of clinical presentation, for these studies, the decision to classify as ALI/ARDS versus CPE was made by clinical expert(s) reviewing clinical information and response to therapy including diuretics. The fundamental questions are: are biomarkers measured at clinical presentation more reliable (and accessible) than post hoc experts’ opinion in differentiating these conditions and can biomarkers identify patients who should have therapy for both conditions (coexisting). Neither question was answered by this systematic review, largely because there is no true gold standard for distinguishing ARDS/ALI from CPE. Given this limitation we understood that the purpose of this review was to identify potential biomarkers that would most closely correlate with expert clinical judgement, which was often post hoc – after the diagnosis became clear. If these biomarkers could be then used for earlier detection and intervention, as suggested by the studies showing that BNP appeared to be most useful when measured at the time of presentation in the emergency department and before initiating therapy, this may allow guidance of appropriate intervention before such time that the clinical differentiation is clear.

## Conclusions

We found that there were no identified biomarkers or tools with high-quality evidence for differentiating ALI/ARDS from CPE. Because there is no objective “gold standard” for diagnosing ALI/ARDS or CPE, a clear distinction between ALI/ARDS and CPE may not have been possible in any of these reported studies. The eventual diagnosis was determined by post hoc expert review, blinded to target marker. These limitations pose an obstacle to developing a reliable method to differentiate these disorders. However, differentiating the cause of pulmonary edema is important because the therapy of ALI/ARDS and CPE are fundamentally different. Although fluid restriction might be used to treat both CPE and ARDS/ALI, early recognition of ALI/ARDS allows an emphasis on lung-protective ventilation and in the treatment of the underlying cause of the ARDS whilst recognition of CPE may lead to the appropriate use of diuretics, inotropic therapy, and afterload reduction. Combining clinical criteria with validated biomarkers may improve the predictive accuracy and improve the outcomes of ALI/ARDS even it co-exists with CPE.

## Abbreviations

ALI: Acute lung injury
ARDS: Acute respiratory distress syndrome
AUC: Area under the curve
BAL: Bronchoalveolar lavage
BNP: Brain natriuretic peptide
CENTRAL: Cochrane Central Register of Controlled Trials
CPE: Cardiogenic pulmonary edema
CRP: C-reactive protein
CT: Computed tomography
ELF: Epithelial lining fluid
IQR: Interquartile range
MOOSE: Meta-analysis of Observational Studies in Epidemiology
PCWP: Pulmonary capillary wedge pressure
PRISMA: Preferred Reporting Items for Systematic Reviews and Meta-Analysis
PVPI: Pulmonary vascular permeability index
SP-A: Surfactant protein-A
SP-B: Surfactant protein-B
